# HIV-Associated Conditions in Older Adults

**DOI:** 10.7759/cureus.32661

**Published:** 2022-12-18

**Authors:** Ecler Jaqua, Wessam Labib, Katalin Danji

**Affiliations:** 1 Family Medicine, Loma Linda University Medical Center, Loma Linda, USA; 2 Geriatrics, Loma Linda University Medical Center, Loma Linda, USA; 3 Geriatrics, Loma Linda University Health, Loma Linda, USA

**Keywords:** hiv drugs, hiv associated neurocognitive disorders, hiv care, hiv diseases, older adult

## Abstract

Geriatric practices will see more people living with human immunodeficiency virus (HIV), as their life expectancy is close to the general population due to effective antiretroviral therapy (ART). Geriatricians focus more on HIV-associated, non-acquired immunodeficiency syndrome (AIDS) disorders than HIV alone. We will review the most common chronic illnesses and conditions associated with aging and HIV. Even though fall frequency in older adults living with HIV is similar to or lower than in people without HIV, fall assessment is appropriate, especially in the high-risk elderly living with HIV. HIV also impacts motor function and memory loss, especially in advanced cases. ART doesn’t cross the blood-brain barrier, leading to major neurocognitive disorders with age. The etiology of HIV and cardiovascular disease (CVD) is multifactorial, including the effect of ART. Pitavastatin and pravastatin cause fewer interactions with ART.* *While the treatment for HIV decreases the risk of opportunistic infections, it may cause several bone-related abnormalities, including low bone mineral density (BMD), osteoporosis, and fractures. Polypharmacy is associated with disability and mortality and may increase the risk of ART drug-drug interaction. The oral health status of HIV-infected patients is commonly inadequate, and the presence of dental care managers may improve clinical outcomes and increase medication adherence. Furthermore, people aging with HIV (PAWH) have an increased mortality risk when co-infected with coronavirus disease 2019 (COVID-19). In summary, older adults living with HIV may face unique challenges. Therefore, providing comprehensive medical care and psychosocial support through an interdisciplinary team can significantly impact their lives.

## Introduction and background

**Figure 1:**
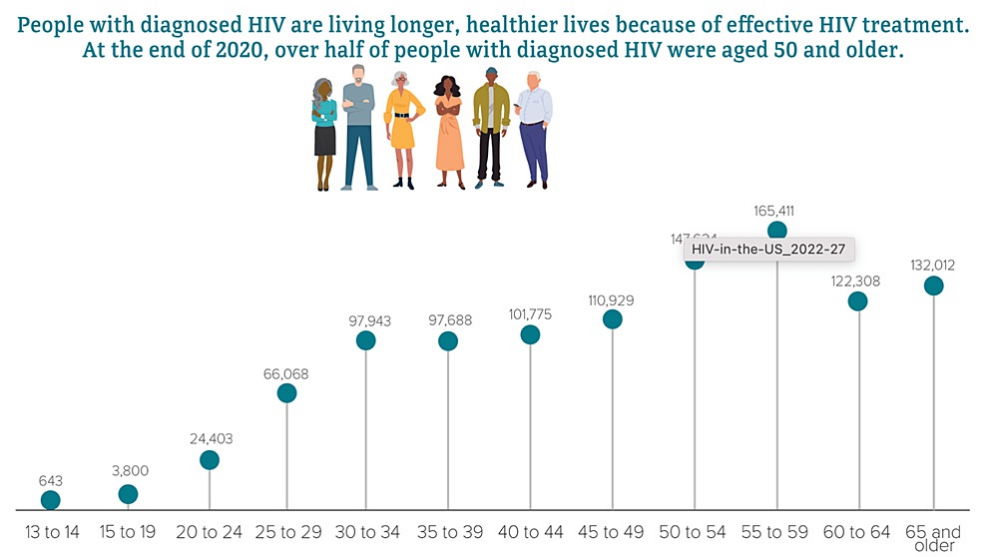
Diagnosis of HIV in the US by Age Source: Centers for Disease Control and Prevention. Diagnoses of HIV infection in the United States and dependent areas, 2020. HIV Surveillance Report 2022;33. https://www.cdc.gov/hiv/library/reports/hiv-surveillance/vol-33/index.html. Published September 2, 2022. Accessed November 21, 2022.

In 2020, 51% or one in six Americans with human immunodeficiency virus (HIV) were 50 years and older, with 17% being new cases (Figure 1) [1]. Individuals in this age group can live healthier and longer lives because of successful HIV treatment. Unfortunately, older adults in the United States (US) are more likely to have late-stage HIV infection at the time of diagnosis when compared with younger individuals because of decreased testing opportunities. Older adults don’t consider themselves a high-risk population to acquire HIV, and healthcare workers may have some misperceptions about sexual activity in this age group [1]. As a result, older adults may start the treatment late, increasing the risk of additional system immune impairment. However, older and young adults usually have the same HIV risk factors such as having multiple partners, lacking knowledge about HIV prevention, and being less likely to use condoms [1,2].

The main focus of healthcare professionals for the aging population with HIV is no longer HIV alone but rather the HIV-associated non-acquired immunodeficiency syndrome (AIDS) disorders, including falls and functional limitations, neurocognitive function, cardiovascular disease, bone loss, oral health, and coronavirus disease 2019 (COVID-19) [1,3,4]. In addition, it is essential to highlight the importance of polypharmacy and medication interaction with HIV therapies since older adults have more chronic diseases and consequently take more medications. Therefore, older adults with HIV should receive continuing assessment and treatment for their comorbidities. Below we will review the most common chronic illnesses and conditions associated with aging and HIV.

## Review

Falls and functional limitations

Current studies showed that fall frequency in older adults living with HIV is similar to or lower than in people without HIV [4,5]. Therefore, targeting the high-risk elderly living with HIV might be an appropriate approach rather than assessing all elderly with HIV. Traditional assessment of falls should be relevant to the elderly living with HIV. Screening for falls at 50 and older has been supported by some studies in patients living with HIV, given the possible comorbidities associated with HIV and falls [5]. Both functional limitations and disability assessments have been utilized in people living with HIV (PLWH). Research in HIV uses the classic geriatric assessment of asking about activities of daily living (ADLs) and instrumental activities of daily living (IADLs) to assess disability. Other studies utilize questions from the Medical Outcomes Study HIV Health Survey (MOS-HIV) or the 36-Item Short Form Health Survey (SF-36), which include self-reports of functional limitations and disability. While self-report of functional limitations is contained in questionnaires like the MOS-HIV, recent emphasis has focused on objective assessments of gait speed, grip strength, chair stands, and the Short Physical Performance Battery (SPPB). The SPPB, in particular, has been used in multiple cross-sectional studies and was recently shown to predict mortality [4,5]. In addition, risk factors for recurrent falls and functional limitations may include frailty, peripheral neuropathy, polypharmacy, low physical activity, and osteoporosis [4,6,7].

Neurocognitive function

Unfortunately, the impact in older patients with HIV on the motor function and memory loss domains is well known, especially in advanced cases, which is usually the circumstance in older adults [3]. Moreover, these two domains are more vulnerable to the progression of major neurocognitive impairment due to differential impairment to the hippocampus and basal ganglia, mainly the nigrostriatal pathways [3,8]. Numerous antiretroviral therapy (ART), such as nelfinavir, ritonavir, and indinavir, doesn’t cross the blood-brain barrier, resulting in the replication of the virus in the nervous system and leading to major neurocognitive disorders as adults of HIV age [8,9]. In addition, HIV-associated neurocognitive disorder (HAND) is multifactorial and can be divided into three different types such as asymptomatic neurocognitive impairment (ANI), mild neurocognitive disorder (MND), and HIV-associated dementia (HAD). Risk factors for HAND may include hyperlipidemia, tobacco use, age > 50 years old, hepatitis C virus infection, and methamphetamine [8,9].

**Figure 2:**
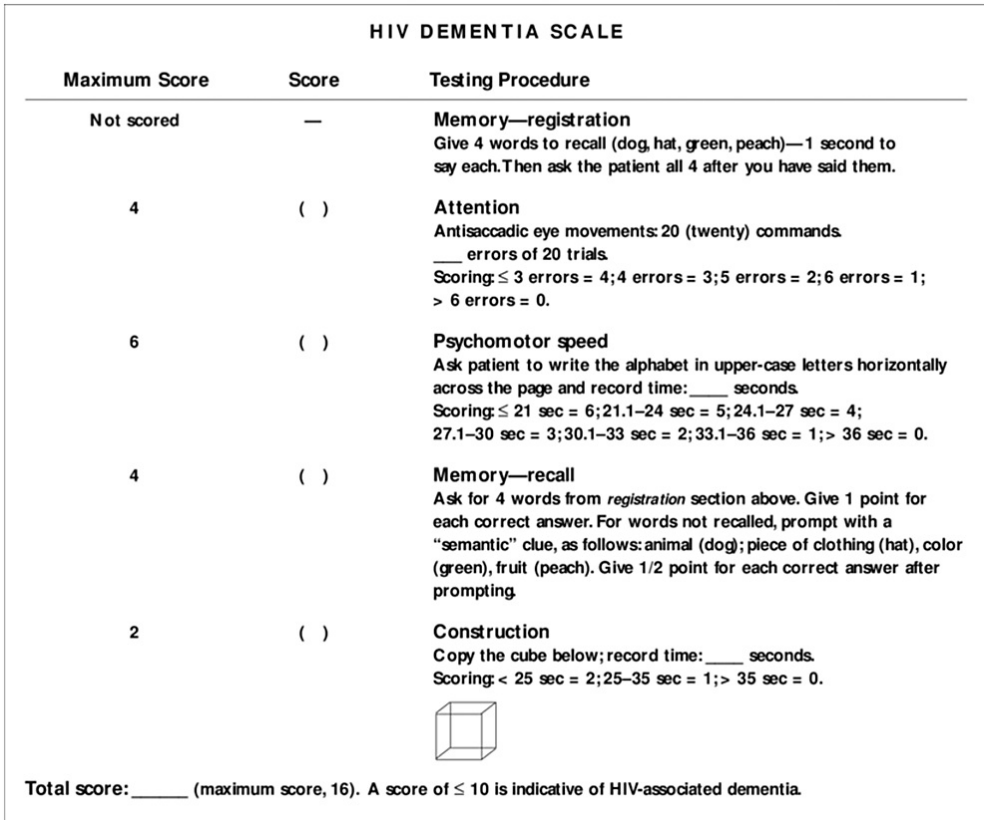
HIV Dementia Scale Catalan J [11]

Although the United States Preventive Services Task Force (USPSTF) reported insufficient evidence to screen older adults for cognitive impairment, Medicare compensates for diagnosing cognitive impairment as part of the annual wellness visit. In addition, a few studies with little evidence showed that early detection of cognitive impairment might help family members and improve outcomes [10]. Screening tools specific to HIV-associated neurocognitive disorder include the HIV dementia scale (Figure 2), Frascati criteria, and Montreal Cognitive Assessment (MoCA) [11,12].

Older adults with HIV infection should also regularly screen for psychosocial habits and substance and alcohol abuse at least annually. More frequent assessments for sleep, eating patterns, and medication interactions are also recommended [3].

Cardiovascular disease

There is an additional cardiovascular disease (CVD) risk in older adults with HIV. The association between HIV and CVD is multifactorial and may include direct viral effects, chronic inflammation, and the effect of medications such as ART [13]. However, the most significant modifiable risk factor for CVD is smoking. Therefore, the American Heart Association (AHA) and American Diabetic Association (ADA) recommend that patients 40 years and older with diabetes and low-density lipoprotein (LDL) above 70 mg/dL be on a statin and aspirin if there are no existent contraindications [13,14]. Pitavastatin and pravastatin have a minimal cytochromes P450 (CYP) metabolism; therefore, it causes fewer interactions with ART. However, atorvastatin and rosuvastatin, the most common statins used, can cause mild to moderate drug-drug interaction with ART [14].

Bone disease

Low bone mineral density (BMD) is prevalent in HIV-infected patients. While recent innovations in HIV treatment decrease the viral load and reduce the risk of opportunistic infections, it may cause several bone-related abnormalities, including low BMD, osteoporosis, osteopenia, osteomalacia, and fractures [15,16]. Osteoporosis can be three times more frequent in HIV-infected patients than in non-HIV subjects [15]. Of all ART classes, protease inhibitors are the principal agents associated with a higher incidence of bone-related diseases [15,16]. Even though the pathogenesis of bone abnormalities in patients with HIV is not fully understood, a possible theory involves apoptosis of the osteoblasts due to impairment of osteoclasts and bone marrow cells secondary to HIV infection, leading to damage of bone maturation. Unfortunately, ART is known to increase bone loss, especially protease inhibitors. However, studies showed that using integrase inhibitors in combination with other ART may improve BMD. It is also helpful to start supplementation with vitamin D and calcium in all individuals with HIV [16]. Therefore, early screening starting at age of 50 and early treatment are crucial to avoid fractures and functional decline, especially in older adults. The recommended screening intervals are similar to those in individuals with no HIV infection.

- T-score of -2 to -2.49 may repeat BMD at 1-2 years

- T-score of -1.01 to 1.99 may repeat at 5 years

- Normal BMD (T-score >-1) an interval of up to 15 years can be used. If there is a new fragility fracture or risk factor for osteoporosis, BMD should be repeated sooner [17].

Polypharmacy

Aging is associated with age-related physiological changes, organ dysfunction, and an increased number of comorbidities. It can result in complex polypharmacy and an increased risk of adverse health outcomes. The common definition of polypharmacy is the concurrent administration of more than five medications. Polypharmacy ranges from 15% to 94% among PLWH aged more than 50 years [18,19]. Interestingly, the prevalence of polypharmacy is not as pronounced in the age group over 75 years as in younger geriatric patients. Likely, this is part of the natural aging process and is related to increased comorbidities. Medications associated with polypharmacy are cardiovascular drugs, anticoagulants, antiplatelets, gastrointestinal agents, hormone replacement therapy, such as estrogen, progestin or combination pill, benzodiazepines, and muscle relaxants [20]. Adverse health outcomes are associated with polypharmacy, including increased hospitalization, falls, frailty, cognitive decline, functional decline, disability, and mortality [21]. Taking care of patients who take multiple drugs is challenging because of drug-drug interaction risk, inappropriate drug use, prescribing cascade, or drug-disease interactions [19,22]. Improper drug use is defined as the risk of adverse events outweighing the benefit of a prescribed drug. It can lead to adverse drug effects and a subsequent prescribing cascade. Specific skills are needed to recognize adverse drug reactions and not interpret them as new diseases.

Unfortunately, few studies address inappropriate prescribing in the elderly. There are only a few tools, e.g., the American Geriatric Society (AGS) Beers Criteria, a screening tool for older person’s prescriptions (STOPP), and a screening tool to alert to right treatment (START) criteria for recognizing wrong drug indications, dosing, treatment duration, and omission of treatment. These studies show inappropriate prescribing is common and may be as high as 52-69 % among elderly PLWH [19]. Medication reconciliation, medication review, and a patient-centered approach are essential to polypharmacy prevention. The patient-centered approach includes considering the risk and benefits of prescribing each medication, care goals, frailty evaluation, and life expectancy. In the future, we need more studies to address polypharmacy, associated drug-drug interactions, inappropriate medication use, and prescribing cascade or drug-disease interactions in elderly PLWH. In addition, deprescribing is an important concept that needs to be studied and implemented in current practices [19,21].

Oral health

Current antiretroviral management increases the survival of HIV patients and, therefore, grows the need for dental care. Oral health may improve the health-related quality of life of HIV patients [23]. Overall, the oral health status of HIV-infected patients is unsatisfactory; therefore, effective intervention programs are needed to prevent and manage dental problems. It is especially critical in older men with low socioeconomic status and those with high-risk behaviors [24]. One-third of people living with HIV have difficulty treating oral lesions caused by a weakened immune system. Untreated oral diseases can affect the ability to consume enough calories and thus cause weight loss and worsening malnutrition [25]. Also, there is a proven association between oral health and lower mental health in HIV-infected patients with depression [23]. Identifying barriers is one of the most critical steps in providing dental care, and they are different depending on the patient’s living situation [24,25].

However, according to the American Dental Association, a lack of case management services is one of the most significant challenges for dental care. A dental care manager is recommended to bridge the gap between the patient and the healthcare professional. In addition, the AIDS institution of the New York state department of health suggests using a case manager as part of a multidisciplinary approach to increase access to oral care [25]. Several studies found that using a medical case manager is associated with a decrease in the number of unmet needs for supportive care, improved clinical outcomes, and increased adherence to medications [23,25]. Asymptomatic HIV patients can be treated as any other dental patient. Although rare, HIV patients may present with immune thrombocytopenia as a complication of HIV infection, but only severe thrombocytopenia leads to excessive bleeding during invasive dental procedures [23].

COVID-19

The Disease Control and Prevention (CDC) announced the first novel COVID-19 case in the United States on January 21, 2020. It is caused by severe acute respiratory syndrome coronavirus 2 (SARS-CoV-2). COVID-19 was declared a pandemic a few months later, on March 11, 2020. Studies showed higher mortality rates among those with advanced age, living in aggregate settings, males, African Americans, and adults with comorbidities [26]. Common comorbid conditions observed are but are not limited to coronary artery disease, hypertension, type 2 diabetes mellitus, cerebrovascular disease, chronic kidney disease, liver disease, asthma, chronic obstructive pulmonary disease, and tobacco use. Furthermore, people aging with HIV (PAWH) have above-average rates of comorbidities that increase their risk of mortality when co-infected with COVID-19 [27]. Besides medical comorbidities, PAWHs are affected by many psychosocial challenges like loneliness, depression, poor social support, cognitive impairment, and functional impairment [28].

COVID-19 public policies further enhanced psychosocial vulnerabilities among PAWH. Mental health impacts cause medication adherence concerns and fewer engagements in taking ART [29]. Lower adherence to ART will lead to adverse physical and cognitive outcomes. Implementing quarantine, social distancing, and community containment measures reduce access to routine HIV care and testing [30]. Healthcare professionals will face the physical, psychosocial, and economic impacts of the HIV and COVID-19 systemic effects among elderly patients living with HIV in the future. Currently, we have no long-term data to understand its full effects on HIV prevention efforts, diagnosis, and treatment outcomes. Evaluation of these factors is necessary to determine the public health consequences of the pandemic stress caused for this vulnerable population. Lessons learned from this pandemic should be incorporated into future patient care and public policies [29,30].

## Conclusions

Living with HIV is challenging, regardless of age group. However, the elderly population may face unique obstacles compared to younger adults, including social isolation, coexisting conditions, polypharmacy, depression, HIV stigma, and misperceptions by healthcare professionals about sexual activity in this age group.

Older adults living with HIV should get regular medical care and monitoring for any signs and symptoms of changes in their HIV and any associated comorbidity related to HIV and aging. Therefore, providing comprehensive medical care and psychosocial support through a multidisciplinary team can significantly impact the lives of our patients and their family members.
